# Impact of GLP‐1 Receptor Agonists on Suicide Behavior: A Meta‐Analysis Based on Randomized Controlled Trials

**DOI:** 10.1111/1753-0407.70151

**Published:** 2025-08-31

**Authors:** Jingqi Chen, Qiufeng Zhang, Qingping Wu, Xiaoming Zhang, Zhiyi Xiang, Sidong Zhu, Tianfu Dai, Yuexiu Si

**Affiliations:** ^1^ The First Affiliated Hospital of Zhejiang Chinese Medical University (Zhejiang Provincial Hospital of Chinese Medicine) Hangzhou China; ^2^ Basic Medical College, Zhejiang Chinese Medical University Hangzhou China

**Keywords:** GLP‐1 RAs, meta‐analysis, obesity, suicidal behavior, type 2 diabetes mellitus

## Abstract

**Background:**

This meta‐analysis aims to assess the association between exposure to glucagon‐like peptide‐1 receptor agonists (GLP‐1 RAs) and the incidence of suicidal behavior in patients with type 2 diabetes mellitus (T2DM)/obesity.

**Methods:**

A comprehensive search of electronic databases, including PubMed, Web of Science, the Cochrane Library, and ClinicalTrials.gov, was conducted from the inception of the databases. The risk ratio (RR) and 95% confidence intervals (95% CI) were calculated.

**Results:**

This meta‐analysis included data from 25 randomized controlled trials (RCTs). The results indicated no significant difference in the incidence of suicidal behavior between the GLP‐1 RA exposure group and the control group (RR = 0.84, 95% CI: 0.54–1.32, *p* = 0.46, *I* ^2^ = 0%). Subgroup analysis showed no significant differences in the incidence of suicidal behavior among participants with T2DM (RR = 0.74), obesity (RR = 1.07), adolescents (RR = 0.91), and adults (RR = 0.84). Additionally, no significant differences were observed between the two groups in any type of suicidal behavior, including suicidal ideation (RR = 1.04), suicide attempts (RR = 0.68), depression‐related suicides (RR = 0.65), and completed suicides (RR = 1.06). There were also no significant differences between the groups for any type of GLP‐1 RA, including dulaglutide (RR = 0.46), exenatide (RR = 0.98), semaglutide (RR = 0.82), lixisenatide (RR = 1.25), and liraglutide (RR = 0.92). No significant differences were observed between the exposure group and control group according to different comparators, including placebo (RR = 0.91) and others (RR = 1.08). All subgroup analyses showed *p*‐values greater than 0.05 (two‐sided tests) and *I* ^2^ values of 0%.

**Conclusion:**

Our findings suggest that there is no significant association between GLP‐1 RA exposure and suicidal behaviors in patients with T2DM or obesity.

## Graphical abstract

This meta‐analysis included data from 25 randomized controlled trials (RCTs) suggesting that there is no significant association between GLP‐1 RA exposure and suicidal behaviors in patients with T2DM or obesity.

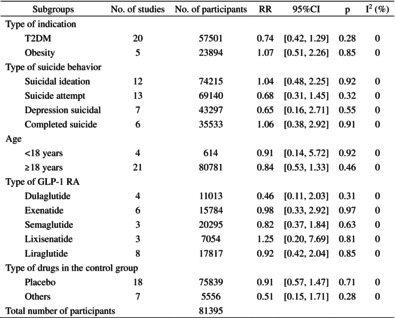

**Summary**

- This meta‐analysis found no significant increase in suicidal behavior risk with GLP‐1 RAs versus controls.
- Results were consistent across populations (T2DM, obesity, adolescents, adults) and outcomes (ideation, attempts, depression‐related, and completed suicides).
- No significant differences were observed between the groups for any type of GLP‐1 RA.
- The findings imply that there is no significant association between GLP‐1 RA exposure and suicidal behaviors in patients with T2DM or obesity.

**Abbreviations**
95% CI: 95% confidence interval
BBB: blood–brain barrier
CCRBT: Cochrane collaboration risk of bias tool
EMA: European Medicines Agency
GLP‐1 RAs: glucagon‐like peptide‐1 receptor agonists
GLP‐1R: glucagon‐like peptide‐1 receptor
HPA: hypothalamic–pituitary–adrenal
IL‐6: interleukin‐6
LPS: lipopolysaccharide
RCTs: randomized controlled trials
RR: risk ratio
T2DM: type 2 diabetes mellitus
TNF‐α: tumor necrosis factor‐alpha

## 1 Introduction

Suicide represents a significant global public health concern. According to global statistics, more than 700 000 individuals die by suicide each year, with many more attempting it. Suicide can occur at any stage of life and has become the third leading cause of death among individuals aged 15–29 [1]. The factors influencing suicide are multifaceted, involving physiological conditions, social environment, and other determinants. Notably, obesity and diabetes are recognized as important risk factors for suicidal behavior.

It is well established that there is a strong correlation between obesity and mental health issues such as depression and anxiety [2]. Individuals with obesity often experience feelings of shame related to their body weight, which can lead to significant psychological distress. Since the onset of the obesity epidemic in the United States, the incidence of suicidal behavior has been found to be higher among obese adolescents compared to their non‐obese peers [3]. Similarly, patients with type 2 diabetes mellitus (T2DM) are also at an increased risk for suicidal tendencies. Many individuals with T2DM suffer from complications such as amputations and cardiovascular diseases, which contribute to a diminished quality of life. A retrospective cohort study found that patients with T2DM had a 1.8‐fold higher risk of developing depressive symptoms compared to individuals without T2DM over a 6.5‐year follow‐up period [4]. Given that depressive symptoms are a known risk factor for suicidal behavior [5], patients with T2DM may be at an elevated risk for suicide.

Glucagon‐like peptide‐1 receptor agonists (GLP‐1 RAs) are widely used therapeutics for managing T2DM and obesity. Theoretically, GLP‐1 RAs could reduce the risk of suicide by improving the management of these conditions. However, reports from the Icelandic Medicines Agency have raised concerns, indicating that some individuals using liraglutide and semaglutide have experienced suicidal thoughts and self‐harm. In response, the European Medicines Agency (EMA) has requested that certain license holders of GLP‐1 RAs provide further evidence regarding suicidal ideation and self‐harm [6].

A previous meta‐analysis examining the relationship between GLP‐1 RAs and mental health, based on randomized controlled trials (RCTs), concluded that no significant association was found between GLP‐1 RAs and mental health outcomes. However, this meta‐analysis had several limitations, including a small sample size of included RCTs and the lack of more detailed subgroup analyses [7]. Therefore, this meta‐analysis aims to comprehensively evaluate the published RCTs to better assess the relationship between GLP‐1 RAs exposure and suicidal behavior in patients with T2DM or obesity.

## 2 Materials and Methods

### 2.1 Research Registration

In accordance with the Meta‐analysis (PRISMA) protocol, this meta‐analysis has been registered in the International Prospective Register of Systematic Reviews (PROSPERO) under registration number CRD42024610002.

### 2.2 Retrieval Strategy

Studies related to GLP‐1 RAs and suicidal behavior were searched in PubMed, Web of Science, and the Cochrane Library from the inception of the respective databases up to October 2024. A comprehensive search strategy was employed, combining both subject headings and free text terms, and all reference lists of the identified articles were screened for additional relevant studies. The following keywords were used in the search: ((GLP‐1 RAs) OR (Glucagon‐like peptide‐1 receptor agonist) OR (semaglutide) OR (liraglutide) OR (exenatide) OR (dulaglutide) OR (lixisenatide) OR (albiglutide)) AND ((suicide) OR (suicide attempt) OR (suicidal ideation) OR (depression‐related suicide) OR (completed suicide)). To minimize the risk of missing relevant studies, a manual search was also conducted on ClinicalTrials.gov to identify completed but unpublished studies.

### 2.3 Data Inclusion and Exclusion Criteria

The inclusion criteria were based on the PICOS framework (Participants, Intervention, Comparison, Outcomes, Study design):

1. Participants with T2DM or obesity.
2. Intervention with GLP‐1 RAs.
3. Comparison with a control group receiving either other hypoglycemic agents, placebo for T2DM, or other weight‐loss medications/placebo for obesity.
4. Outcomes including suicide‐related adverse events.
5. Study design consisting of RCTs.

Studies were excluded based on the following criteria:

1. Non‐English language publications.
2. Unavailable data.
3. Incomplete texts (e.g., abstracts only).
4. Lack of relevant outcomes.
5. When multiple studies from the same cohort were identified, the most comprehensive or recent study was preferentially included.

### 2.4 Data Extraction and Quality Evaluation

Data extraction was performed using a pre‐designed table to capture study details (authors, publication year, study design, clinical trial registration number, etc.), participant characteristics (gender, age, treatment regimen, health status, etc.), and suicide‐related outcomes.

The quality of each study was assessed by two independent reviewers. Risk of bias was evaluated using the Cochrane collaboration risk of bias tool (CCRBT) for RCTs, following Cochrane guidelines. The assessment was based on the following seven domains:

1. Random sequence generation (selection bias).
2. Allocation concealment (selection bias).
3. Blinding of participants and personnel (performance bias).
4. Blinding of outcome assessment (detection bias).
5. Incomplete outcome data (attrition bias).
6. Selective reporting (reporting bias).
7. Other sources of bias.

Each domain was classified as “low risk,” “high risk,” or “unclear risk.” Disagreements between reviewers were resolved through discussion with a third reviewer to reach a consensus.

### 2.5 Statistical Analysis

Statistical analysis was performed using Review Manager 5.3 (RevMan version 5.3; Oxford, UK). The risk ratio (RR) and the 95% confidence interval (95% CI) were calculated to assess the association between GLP‐1 RAs and the incidence of suicide‐related adverse events. An RR value of less than 1 indicates a lower incidence of suicide in the GLP‐1 RA‐exposed group, while an RR greater than 1 suggests a higher incidence in the control group. Heterogeneity across studies was assessed using the chi‐square test, and the inconsistency index (*I* ^2^) was calculated. The following thresholds were applied to interpret *I* ^2^: *I* ^2^ ≤ 25% was considered no heterogeneity, *I* ^2^ > 25% and *I* ^2^ ≤ 50% indicated mild heterogeneity, *I* ^2^ > 50% and *I* ^2^ ≤ 75% indicated moderate heterogeneity, and *I* ^2^ > 75% indicated severe heterogeneity. All analyses used random‐effects models prespecified to address clinical heterogeneity across studies. Additionally, publication bias was assessed using Egger's test. All statistical tests were two‐sided, and statistical significance was set at *p* < 0.05.

## 3 Results

### 3.1 Description of Study Retrieval

**FIGURE 1:**
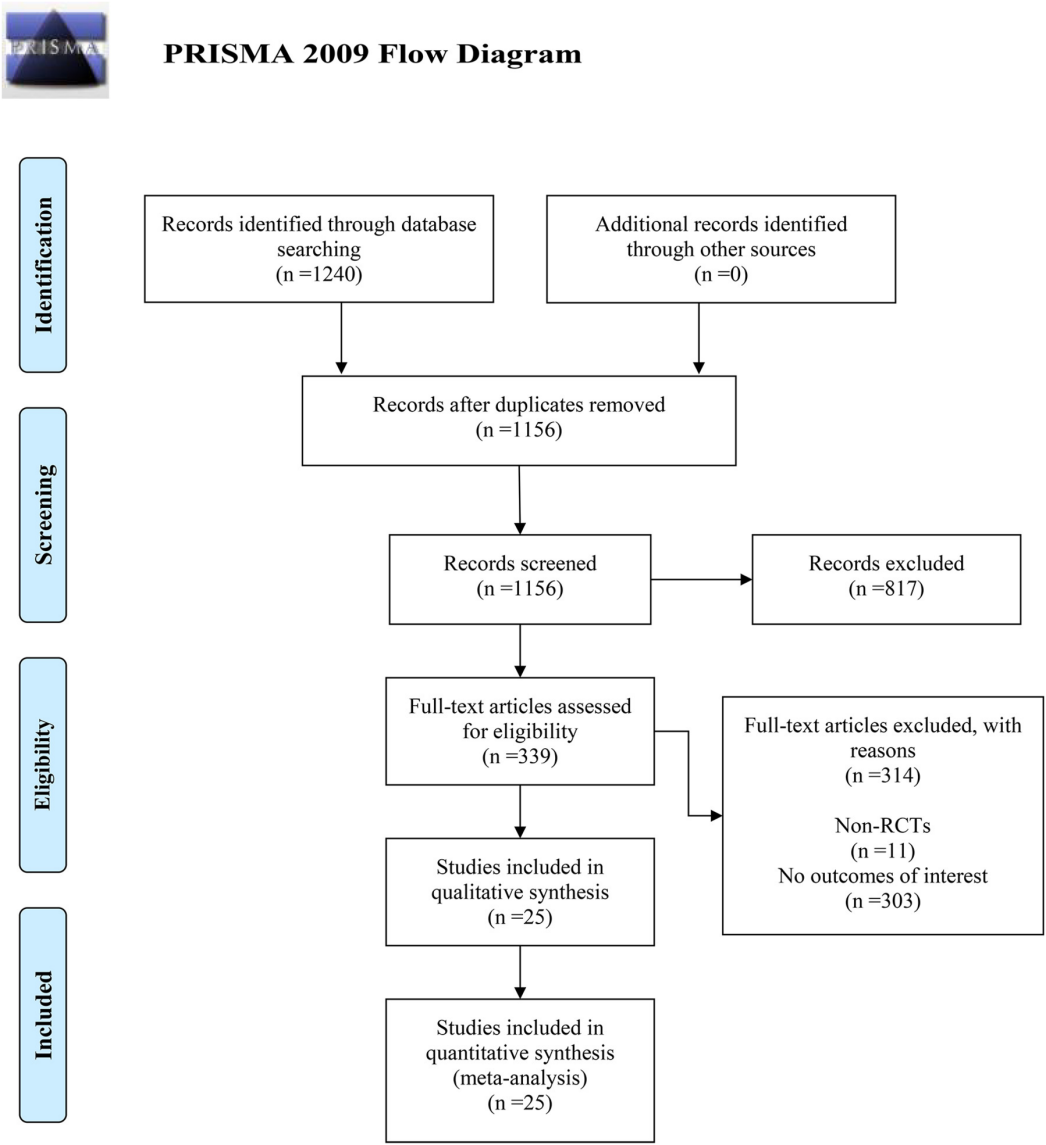
Flow diagram illustrating the study selection process.

A systematic search yielded 1240 records from four electronic databases. After removing duplicates, 1156 studies remained. Titles and abstracts were then screened, resulting in the exclusion of 817 studies, leaving 339 studies for further assessment. Following full‐text review, 11 studies were excluded due to RCT designs, and 303 studies were excluded for lacking relevant outcomes. Ultimately, a total of 25 studies [8, 9, 10, 11, 12, 13, 14, 15, 16, 17, 18, 19, 20, 21, 22, 23, 24, 25, 26, 27, 28, 29, 30, 31, 32] met the inclusion criteria and were included in the meta‐analysis, as illustrated in Figure 1.

### 3.2 Characteristics of Studies and Participants

The 25 RCTs [8, 9, 10, 11, 12, 13, 14, 15, 16, 17, 18, 19, 20, 21, 22, 23, 24, 25, 26, 27, 28, 29, 30, 31, 32], published between 2010 and 2023, collectively enrolled over 81 000 participants. Of these, more than 42 000 participants were assigned to the GLP‐1 RAs exposure group, while more than 39 000 participants were in the control group. Among these studies, 20 involved participants with T2DM, and 5 involved participants with obesity (non‐T2DM). All participants in the exposure group received GLP‐1 RAs for the treatment of T2DM or obesity. In the control group, T2DM participants received either placebo or other hypoglycemic agents, including metformin, insulin, and alpha‐glucosidase inhibitors, while participants with obesity received only placebo. Additionally, four studies included adolescents (< 18 years), while the remaining 21 studies enrolled adults. Detailed study characteristics are provided in Table 1, and further participant details are available in Table S1.

**TABLE 1:** Characteristics of the studies included in the meta‐analysis.

Author	Year	Clinicaltrials.gov number	Indication	Number of patients		Treatment regimens	
				Experiment	Control	Experiment	Control
Umpierrez, G.	2014	NCT01126580	T2DM	539	268	Dulaglutide 0.75/1.5 mg	Metformin
Gerstein, H.C.	2019	NCT01394952	T2DM	4943	4949	Dulaglutide 1.5 mg	Placebo
Mentz, R.J.	2017	NCT01144338	T2DM	7344	7372	Exenatide 2 mg	Placebo
Zinman, B.	2019	NCT03021187	T2DM	546	184	Semaglutide 3/7/14 mg	Placebo
Tamborlane, W.V.	2022	NCT01554618	T2DM	59	23	Exenatide 2 mg	Placebo
Hernandez, A.F.	2018	NCT02465515	T2DM	4717	4715	Albiglutide 30/50 mg	Placebo
Ahrén, B.	2013	NCT00712673	T2DM	255	85	Lixisenatide 10 mg	Placebo
Jabbour, S.A.	2020	NCT02229396	T2DM	231	233	Exenatide + Dapagliflozin	Dapagliflozin + Placebo
Pfeffer, M.A.	2015	NCT01147250	T2DM	3031	3032	Lixisenatide 20 mg	Placebo
Unger, J.	2021	NCT02730377	T2DM	980	984	Liraglutide 1.8 mg	Oral Antidiabetic Drug
Kaku, K.	2019	NCT02607306	T2DM	273	546	Liraglutide 1.8 mg	Insulin OR Insulin + Liraglutide 0.36 mg
Seino, Y.	2012	NCT00866658	T2DM	154	157	Lixisenatide 20 mg	Placebo
DeFronzo, R.A.	2010	NCT00135330	T2DM	92	45	Exenatide + Rosiglitazone OR Exenatide	Rosiglitazone
Arslanian, S.A.	2022	NCT02963766	T2DM	104	51	Dulaglutide 0.75/1.5 mg	Placebo
Ishii, H.	2020	NCT02750410	T2DM	120	39	Dulaglutide 0.75 mg	Placebo
Buse, J.B.	2011	NCT00765817	T2DM	137	122	Exenatide 5/10 mg	Placebo
Seino, Y.	2012	NCT00393718	T2DM	268	132	Liraglutide 0.9 mg	Glibenclamide
Tack, C.J.	2019	NCT01179048	T2DM	4668	4672	Liraglutide 1.2 mg	Placebo
None	2014	NCT01117350	T2DM	481	484	Liraglutide 1.8 mg	Insulin Glargine
Carydias, E.	2022	NCT00658021	T2DM	84	42	Exenatide 5/10 mg	Placebo
Lincoff, A.M.	2023	NCT03574597	Obesity	8803	8801	Semaglutide	Placebo
Pi‐Sunyer, X.	2015	NCT01272219	Obesity	2418	1242	Liraglutide 3.0 mg	Placebo
Wilding, J.P.H.	2021	NCT03548935	Obesity	1306	655	Semaglutide 2.4 mg	Placebo
Kelly, A.S.	2020	NCT02918279	Obesity	125	126	Liraglutide 3.0 mg	Placebo
Blackman, A.	2016	NCT01557166	Obesity	176	179	Liraglutide 3.0 mg	Placebo

Abbreviation: T2DM, type 2 diabetes mellitus.

### 3.3 Quality Evaluation and Risk of Bias

The quality assessment results are presented in Figures S1 and S2. All studies were evaluated using CCRBT and met the requirements for inclusion in the meta‐analysis. Four studies were classified as having a high risk of bias due to the lack of blinding for participants and personnel, as a result of non‐stratified randomization. Three studies were deemed to have a high risk of detection bias due to unblinded outcome assessment. Overall, the majority of studies were classified as low risk for bias, and all studies were considered to be of high quality.

### 3.4 Analysis of Main Outcome and Publication Bias

**FIGURE 2:**
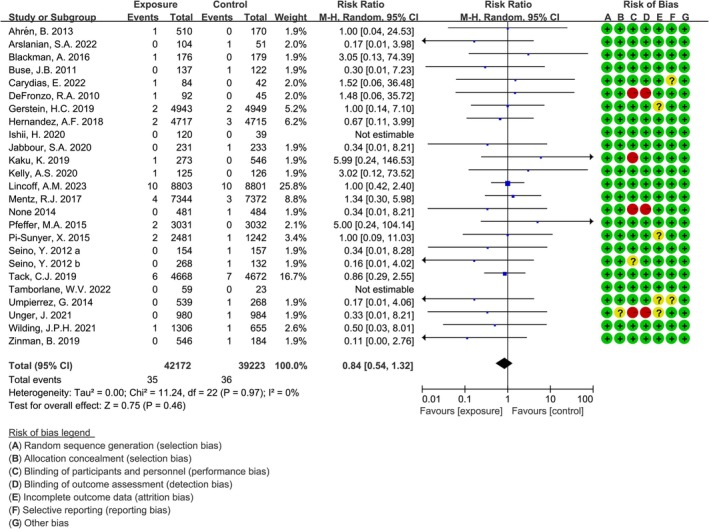
Forest plot depicting the association between GLP‐1 RAs exposure and suicidal behavior in participants with T2DM or obesity.

**FIGURE 3:**
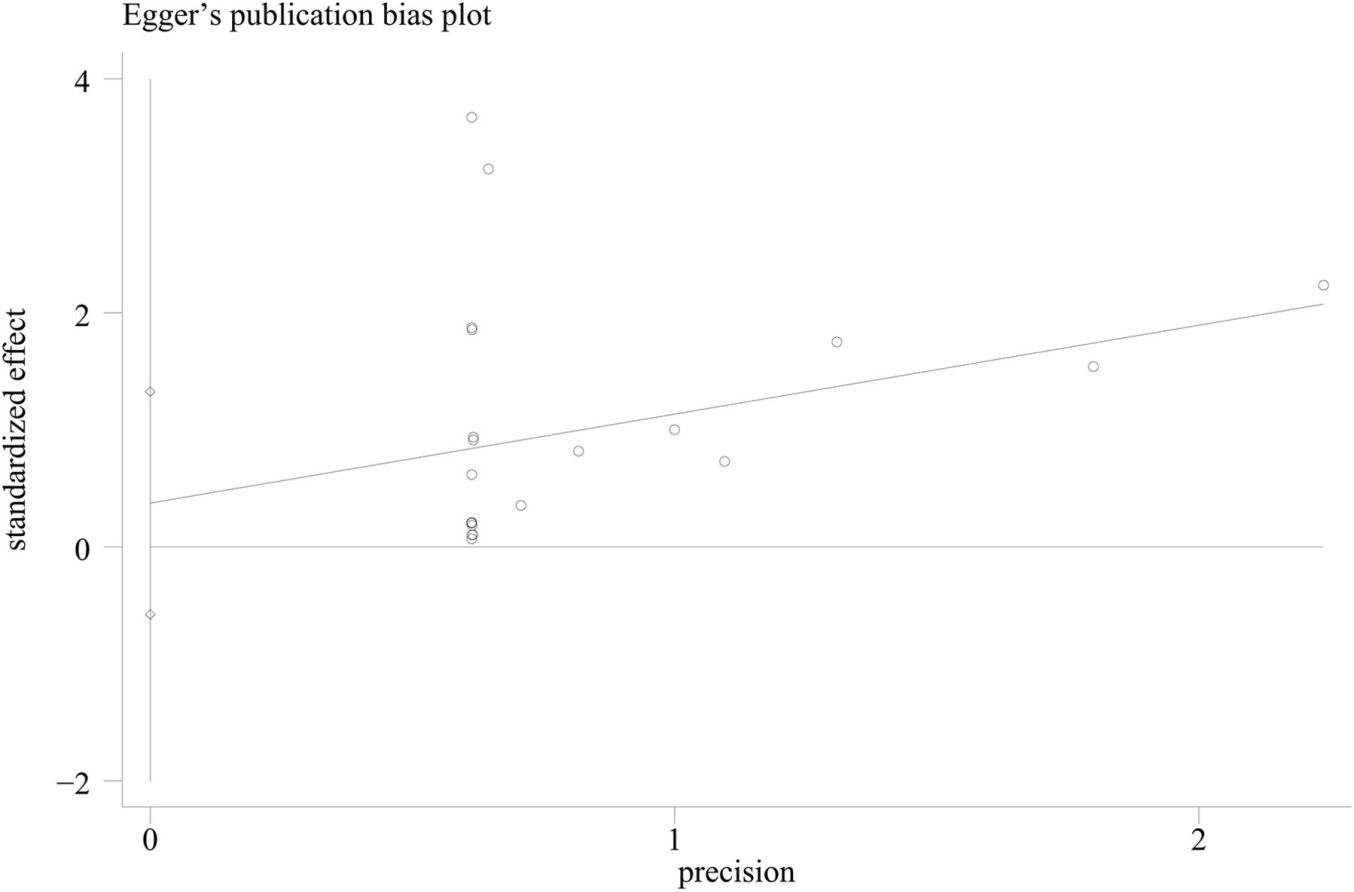
Egger's publication bias plot in the meta‐analysis (*p* = 0.423).

The 25 studies evaluated the association between GLP‐1 RAs exposure and suicide behavior. The pooled analysis revealed no significant difference between the GLP‐1 RAs exposure group and the control group (RR = 0.84, 95% CI: 0.54–1.32, *p* = 0.46, *I* ^2^ = 0%; Figure 2). To investigate the association between GLP‐1 RAs and different suicide behaviors, these events were further categorized into suicidal ideation, suicidal attempts, depression‐related suicide, completed suicide, and self‐injury. The analysis revealed no significant differences between the two groups for any type of suicide behavior (suicidal ideation: RR = 1.04, *p* = 0.92; suicidal attempt: RR = 0.68, *p* = 0.32; depression‐related suicide: RR = 0.65, *p* = 0.55; completed suicide: RR = 1.06, *p* = 0.91). To assess publication bias in the studies examining the relationship between exposure to GLP‐1 RAs and suicide behavior, Egger's test was employed. The analysis revealed no significant publication bias (*p* = 0.423), as depicted in Figure 3.

### 3.5 Subgroup Analysis

Based on participant indication, the studies were divided into two subgroups: patients with T2DM and those with obesity. The statistical results indicated no significant difference in the incidence of suicide behaviors between the two groups, with T2DM participants (RR = 0.74, *p* = 0.28) and obesity participants (RR = 1.07, *p* = 0.85). Among adolescents (< 18 years) in four RCTs, there was no statistically significant difference in the incidence of suicide behaviors between the two groups (RR = 0.91, *p* = 0.92). Similarly, among adults in 21 RCTs, the difference in suicide behavior incidence between the two groups was also not statistically significant (RR = 0.84, *p* = 0.46). Detailed results are presented in Table 2.

**TABLE 2:** Subgroup analysis of the association between GLP‐1 RA exposure and suicidal behavior in participants with T2DM or obesity.

Subgroups	No. of studies	RR	95% CI	*p*	*I* ^2^ (%)
Type of indication					
T2DM	20	0.74	[0.42, 1.29]	0.28	0
Obesity	5	1.07	[0.51, 2.26]	0.85	0
Type of suicide behavior					
Suicidal ideation	12	1.04	[0.48, 2.25]	0.92	0
Suicide attempt	13	0.68	[0.31, 1.45]	0.32	0
Depression suicidal	7	0.65	[0.16, 2.71]	0.55	0
Completed suicide	6	1.06	[0.38, 2.92]	0.91	0
Age					
< 18 years	4	0.91	[0.14, 5.72]	0.92	0
≥ 18 years	21	0.84	[0.53, 1.33]	0.46	0
Type of GLP‐1 RA					
Dulaglutide	4	0.46	[0.11, 2.03]	0.31	0
Exenatide	6	0.98	[0.33, 2.92]	0.97	0
Semaglutide	3	0.82	[0.37, 1.84]	0.63	0
Lixisenatide	3	1.25	[0.20, 7.69]	0.81	0
Liraglutide	8	0.92	[0.42, 2.04]	0.85	0
Type of drugs in the control group					
Placebo	18	0.91	[0.54, 1.53]	0.72	0
Others	7	1.08	[0.40, 2.98]	0.88	0

Abbreviations: 95% CI, 95% confidence intervals; GLP‐1 RA, glucagon‐like peptide‐1 receptor agonists; No., Number; RR, risk ratio; T2DM, type 2 diabetes mellitus.

Additionally, when grouped by GLP‐1 RA type, no significant differences were found between the two groups for any of the drugs (dulaglutide: RR = 0.46, *p* = 0.31; exenatide: RR = 0.98, *p* = 0.97; semaglutide: RR = 0.82, *p* = 0.63; lixisenatide: RR = 1.25, *p* = 0.81; liraglutide: RR = 0.92, *p* = 0.85). No significant differences were observed between the exposure and control groups depending on the control drug type (placebo: RR = 0.91, *p* = 0.72; others: RR = 1.08, *p* = 0.88). All subgroup analyses showed no heterogeneity. Further details are provided in Table 2.

## 4 Discussion

This meta‐analysis synthesized evidence from 25 RCTs to evaluate the potential association between GLP‐1 RAs and suicide‐related behaviors. Our findings demonstrate no significant correlation between the use of GLP‐1 RAs and suicidal behavior in patients with T2DM or obesity.

Obesity is an independent risk factor for suicidal behavior. Individuals with obesity often face societal stigma, discrimination, and social exclusion due to poor body image, which can lead to low self‐esteem and social dysfunction. These societal pressures may exacerbate psychological distress, subsequently elevating the risk of suicide. Furthermore, excessive circulating adipokines in obese individuals promote the increased expression of inflammatory mediators [33], potentially contributing to a chronic low‐grade inflammatory state. These inflammatory mediators may cross the blood–brain barrier (BBB), disrupting the metabolism and function of neurotransmitters such as serotonin and dopamine, which are essential for emotional and behavioral regulation. Additionally, chronic inflammation activates the hypothalamic–pituitary–adrenal (HPA) axis. Prolonged exposure to glucocorticoids can induce glucocorticoid resistance, stimulate immune cell activation, and enhance the production of inflammatory mediators, thus exacerbating neurotoxic effects. Concurrently, chronic glucocorticoid exposure has been shown to cause neuronal damage and loss in structures of the limbic system (e.g., hippocampus and amygdala), potentially increasing the susceptibility to suicidal behavior [34].

Patients with T2DM exhibit higher rates of depression and suicide compared to the general population [35]. This elevated suicide risk stems from a combination of physiological, psychological, social, and nutritional factors [36]. Similar to individuals with obesity, T2DM patients often experience chronic inflammatory states, which may contribute to depression and suicidal tendencies [37]. Animal studies have shown that T2DM mouse models exhibit hippocampal neuronal loss and atrophy [38]. Given that the hippocampus plays a critical role in emotional processing and memory formation, structural deficits in this region correlate with depressive symptoms, such as food avoidance and behavioral immobilization in murine models. These findings suggest that T2DM may predispose individuals to psychiatric disorders, including depression and suicidality, through neurological dysfunction [39]. Psychosocially, T2DM patients face multiple stressors, including diabetes‐related anxieties (e.g., fear of complications), the burdens of disease management (e.g., daily glucose monitoring, insulin injections, and dietary restrictions), as well as financial challenges and insufficient social support—each of which is a potential suicide risk factor [4].

A study has proposed a potential mechanism linking GLP‐1 RAs to suicide risk through their hypothalamic effects. During obesity treatment, GLP‐1 RAs bind to GLP‐1R in the hypothalamus, brainstem, and septal nuclei, particularly regulating feeding behavior in the brainstem and hypothalamic regions [40]. Activation of GLP‐1R may increase the expression of interleukin‐6 (IL‐6) in the brainstem and elevate IL‐6/IL‐1β levels in the hypothalamus [41]. Although these inflammatory mediators suppress appetite and promote weight loss, their overexpression could theoretically increase the risk of suicide. Furthermore, hypothalamic overactivation induced by GLP‐1 RAs may disrupt stress regulation through hyperactivation of the HPA axis, potentially leading to hippocampal dysfunction, cognitive deficits, and suicidal behavior, especially in patients with coexisting psychosocial risk factors [42].

Moreover, acute central administration of GLP‐1 RAs activates GLP‐1R in the raphe nucleus, directly inducing anxiety‐like behaviors. This pharmacological intervention also increases the expression of serotonin (5‐HT2A and 5‐HT2C) receptors and serotonin turnover in the amygdala [43]. Animal studies show that overexpression of the 5‐HT2C receptor in the limbic system increases anxiety‐like behaviors in mice [44]. These pathways may contribute to the induction of negative emotions and increase suicide risk.

Paradoxically, chronic GLP‐1 RA administration demonstrates antidepressant effects that may reduce suicidal behavior. Animal models indicate that exenatide and liraglutide exert antidepressant effects in T2DM mice and antipsychotic‐treated rats, respectively [45, 46]. Notably, the blockade of 5HT2A/5HT2C receptors does not affect the antidepressant efficacy of GLP‐1 RAs [47], suggesting that distinct emotional pathways are activated during acute versus chronic GLP‐1R activation. Therefore, patients may be more likely to benefit from the antidepressant effects of GLP‐1 RAs in long‐term anti‐obesity or anti‐T2DM treatments. Emerging evidence suggests that GLP‐1 RAs may mitigate suicide risk through anti‐inflammatory mechanisms, including direct and indirect immunomodulation via suppression of myeloid cell‐driven inflammation (monocytes/macrophages). Additionally, GLP‐1R activation reduces the induction of plasma tumor necrosis factor‐alpha (TNF‐α) by various Toll‐like receptor agonists, such as lipopolysaccharide (LPS) [48]. Furthermore, following GLP‐1R activation, anti‐inflammatory effects are mediated via GPCR signaling: Gαs‐mediated cAMP elevation activates the Epac/PKA pathways, while PI3K/Akt‐CREB signaling enhances Bcl‐2/Bcl‐xL expression and inhibits pro‐apoptotic proteins (BAD/p53) [49]. Collectively, these mechanisms reduce neuroinflammation, protect neural cells from oxidative stress, and restore neurotransmitter balance.

The aforementioned studies have demonstrated that GLP‐1R is expressed in brain regions involved in emotion regulation, such as the hypothalamus and limbic system. Upon activation of GLP‐1R, GLP‐1 RAs can influence affective states through various mechanisms. However, our meta‐analysis suggests that these pathways do not translate into clinically significant behavioral risks in humans.

Although obese participants may theoretically experience greater psychosocial stress than those with T2DM, our subgroup analyses revealed no significant differences in the incidence of suicide‐related behaviors between GLP‐1 RA exposure groups and control groups in either population. Adolescents, in particular, are more susceptible to social exclusion, discrimination, and stigmatization compared to adults. Moreover, trauma and abuse during sensitive developmental periods can impact cortical and subcortical development, directly influencing suicidal thoughts, behaviors, and self‐harm tendencies in adolescents. Previous studies have shown that suicidal ideation is prevalent among adolescents, with a prevalence rate ranging from 15% to 25% [50]. Despite these factors, our study conducted a subgroup analysis based on age and found no significant differences between the exposed group and the control group, regardless of whether participants were adolescents or adults.

A study has suggested that liraglutide exhibits relatively poor permeability across the BBB, whereas lixisenatide has been shown to cross the BBB and exert neuroprotective effects [49]. Consequently, it is conceivable that different types of GLP‐1 RAs may have varying impacts on the central nervous system. However, our subgroup analyses revealed no significant differences between the exposure and control groups for any of the GLP‐1 RA types. Additionally, no intergroup variations were observed for specific subtypes of suicidal behavior. No significant differences were observed between the exposure group and control group when comparing placebo or others, potentially due to interstudy participant heterogeneity and uncertain definitions of suicide behavior events. The underlying mechanisms require further investigation.

Our meta‐analysis is strengthened by large sample sizes, high‐quality RCTs, and low heterogeneity. Furthermore, the comprehensive subgroup analyses systematically demonstrate that there is no significant association between GLP‐1 RAs and suicidal behavior in populations with T2DM or obesity. However, certain limitations should be considered when interpreting these results. Despite the substantial sample sizes, statistical power remains limited for rare outcomes, such as suicidal behaviors. Low‐incidence events are inherently susceptible to multifactorial confounding, which may impact the precision of our findings.

## 5 Conclusion

In conclusion, no significant correlation was observed between GLP‐1 RAs exposure and suicidal behaviors, regardless of whether the participants had T2DM or obesity. Subgroup analyses, stratified by indication, age, type of GLP‐1 RAs, and specific subtypes of suicidal behaviors, further confirmed the absence of such a correlation.

## Author Contributions

**Jingqi Chen:** conceptualization, methodology, writing – original draft. **Qiufeng Zhang:** conceptualization, writing – original draft. **Qingping Wu:** data curation, methodology. **Xiaoming Zhang:** data curation. **Zhiyi Xiang:** software, validation. **Sidong Zhu:** software; visualization. **Tianfu Dai:** writing – review and editing. **Yuexiu Si:** writing – review and editing.

## Ethics Statement

The authors have nothing to report.

## Consent

The authors have nothing to report.

## Conflicts of Interest

The authors declare no conflicts of interest.

## Supporting information


**Figure S1:** Risk of bias graph used for quality evaluation in the meta‐analysis.

**Figure S2:** Risk of bias summary for quality evaluation in the meta‐analysis.

**Table S1:** Characteristics of the studies included in the meta‐analysis.

## Data Availability

The datasets supporting the conclusions of this article are included within the article itself. For further details, please contact the corresponding author.
