# Supplementary figures and images for: Impact of GLP‐1 Receptor Agonists on Suicide Behavior: A Meta‐Analysis Based on Randomized Controlled Trials
Source: J Diabetes. 2025 Aug 31;17(9):e70151. doi: 10.1111/1753-0407.70151 (PMC12399406; doi:10.1111/1753-0407.70151)

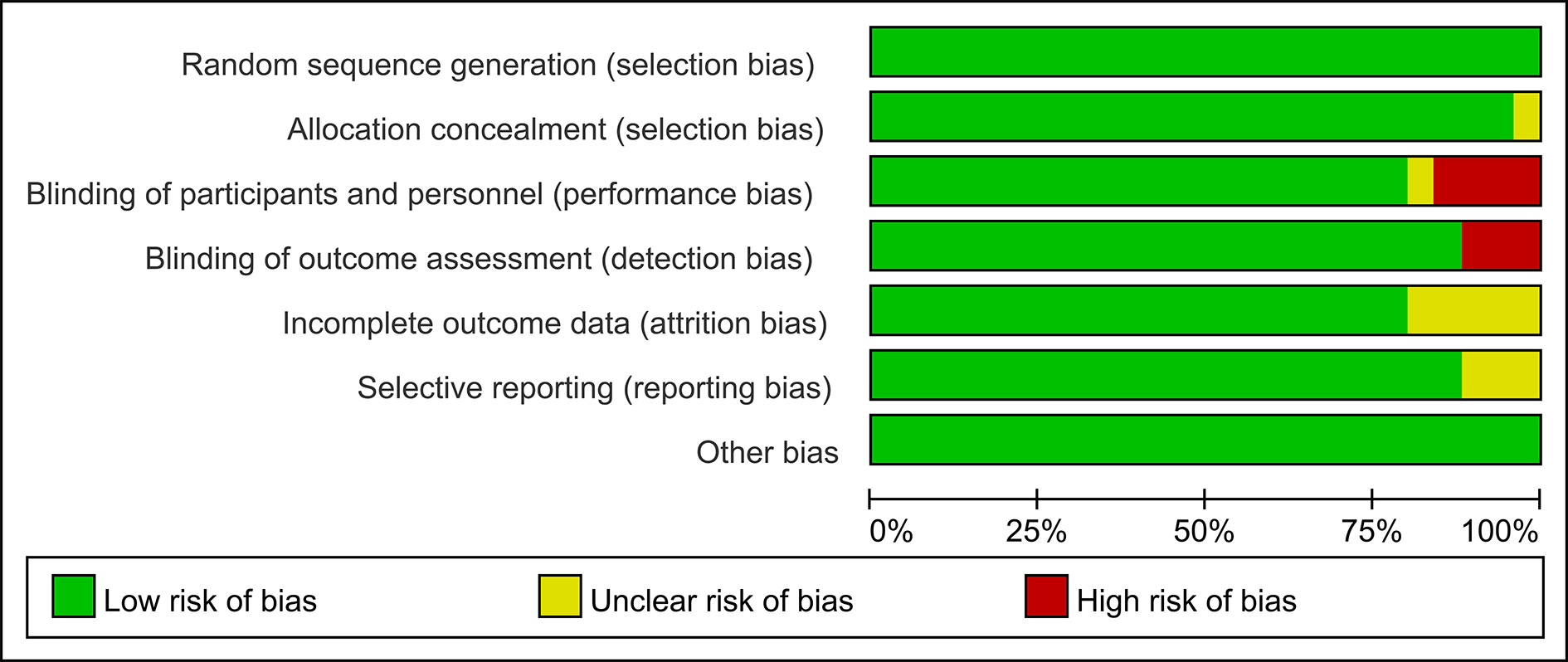

Supplement: Supplementary file 1 — Figure S1: Risk of bias graph used for quality evaluation in the meta‐analysis. [file JDB-17-e70151-s002.tif]

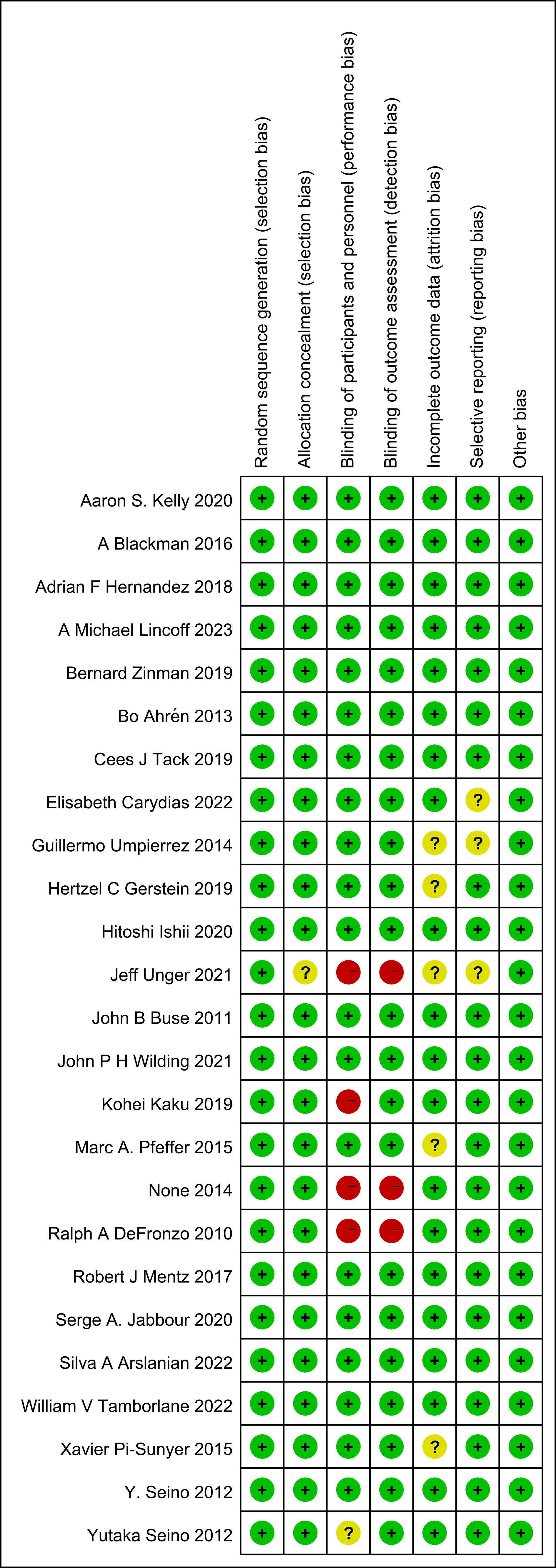

Supplement: Supplementary file 2 — Figure S2: Risk of bias summary for quality evaluation in the meta‐analysis. [file JDB-17-e70151-s001.tif]
