# Supplementary material for: Impact of GLP‐1 Receptor Agonists on Suicide Behavior: A Meta‐Analysis Based on Randomized Controlled Trials
Source: J Diabetes. 2025 Aug 31;17(9):e70151. doi: 10.1111/1753-0407.70151 (PMC12399406; doi:10.1111/1753-0407.70151)
Supplement: Supplementary file 3 — Table S1: Characteristics of the studies included in the meta‐analysis. [file JDB-17-e70151-s003.doc]

Supplementary Table 1. Characteristics of all the studies included in the meta-analysis.

| Author | Year | Age  Mean ± SD (years) | Male  (%) | BMI  Mean ± SD  (kg/m^2) | HbA1c  Mean ± SD  (%) | Duration of diabetes  Mean ± SD (years) |
| --- | --- | --- | --- | --- | --- | --- |
| Umpierrez, G. | 2014 | 55.56 ± 10.38 | 43.7 | 33.26 ± 5.53 | 7.61 ± 0.89 | 2.63 ± 1.83 |
| Gerstein, H.C. | 2019 | 66.2 ± 6.5 | 53.7 | N/A | N/A | N/A |
| Mentz, R.J. | 2017 | 61.9 ± 9.4 | 62.0 | N/A | N/A | N/A |
| Zinman, B. | 2019 | 61 ± 10 | 54.0 | N/A | 8.2 ± 0.7 | N/A |
| Tamborlane, W.V. | 2022 | 15.1 ± 1.84 | 41.5 | N/A | N/A | N/A |
| Hernandez, A.F. | 2018 | 64.1 ± 8.68 | 69.4 | N/A | N/A | N/A |
| Ahrén, B. | 2013 | 54.7 ± 9.7 | 43.1 | 32.91 ± 6.36 | 8.06 ± 0.89 | 6.11 ± 5.17 |
| Jabbour, S.A. | 2020 | 54.2 ± 9.53 | 47.9 | N/A | N/A | N/A |
| Pfeffer, M.A. | 2015 | 60.3 ± 9.7 | 69.3 | 30.16 ± 5.69 | 7.68 ± 1.30 | 9.29 ± 8.25 |
| Unger, J. | 2021 | 57.4 ± 10.8 | 52.4 | N/A | N/A | N/A |
| Kaku, K. | 2019 | 57.2 ± 10.1 | 70.9 | N/A | 8.45 ± 1.06 | N/A |
| Seino, Y. | 2012 | 58.4 ± 10.2 | 47.9 | 25.26 ± 3.82 | 8.53 ± 0.76 | 13.92 ± 7.71 |
| DeFronzo, R.A. | 2010 | 55.70 ± 10.09 | 51.1 | N/A | N/A | N/A |
| Arslanian, S.A. | 2022 | 14.50 ± 2.04 | 28.6 | N/A | 8.08 ± 1.26 | N/A |
| Ishii, H. | 2020 | 59.3 ± 10.31 | 61.6 | N/A | 8.53 ± 0.70 | N/A |
| Buse, J.B. | 2011 | 59.01 ± 9.41 | 57.1 | N/A | N/A | N/A |
| Seino, Y. | 2012 | 58.3 ± 10.4 | 67.3 | 24.81 ± 3.74 | 8.28 ± 0.76 | 8.25 ± 6.73 |
| Tack, C.J. | 2019 | 64.3 ± 7.2 | 64.3 | N/A | N/A | N/A |
| None | 2014 | 57.25 ± 8.81 | 54.3 | 31.88 ± 4.18 | 9.07 ± 1.09 | N/A |
| Carydias, E. | 2022 | 14.0 ± 1.91 | 32.8 | N/A | N/A | N/A |
| Lincoff, A.M. | 2023 | 61.6 ± 8.9 | 72.3 | N/A | N/A | N/A |
| Pi-Sunyer, X. | 2015 | 45.1 ± 12.0 | 21.5 | 38.3 ± 6.4 | 5.6 ± 0.4 | N/A |
| Wilding, J.P.H. | 2021 | 46 ± 13 | 25.9 | N/A | N/A | N/A |
| Kelly, A.S. | 2020 | 14.5 ± 1.6 | 40.6 | N/A | N/A | N/A |
| Blackman, A. | 2016 | 48.5 ± 9.7 | 71.9 | 39.1 ± 6.9 | 5.7 ± 0.4 | N/A |

SD, Standard deviation; BMI, Body mass index; HbA1c, Glycosylated hemoglobin; N/A, Not applicable.
